# Guided Growth as a Component of Joint-Preserving Management of Complex Osteoarticular Pathology in the Growing Knee: A Pediatric Case Report

**DOI:** 10.3390/children13101312

**Published:** 2026-09-26

**Authors:** Kyeong Baek Kim, Jung Shin Kim, Jae Min Cho, Won Chul Shin, Min Uk Do, Jung Yun Bae, Sang-Min Lee

**Affiliations:** 1 Department of Orthopedic Surgery, Research Institute for Convergence of Biomedical Science and Technology, Pusan National University Yangsan Hospital, 20 Geumo-ro, Mulgeum-eup, Yangsan-si 50612, Gyeongsangnam-do, Republic of Korea; pnuyh.102@gmail.com (K.B.K.); whwoals0505@naver.com (J.M.C.); dreami3e5t@pusan.ac.kr (W.C.S.); do7613@naver.com (M.U.D.); nari1006@gmail.com (J.Y.B.); 2 Pusan National University, School of Medicine, Yangsan-si 50612, Gyeongsangnam-do, Republic of Korea; 3 Department of Orthopedic Surgery, College of Medicine, Dong-A University, Busan 49201, Republic of Korea; pnuyh.kjs@gmail.com; 4 Shiley Center for Orthopaedic Research and Education at Scripps Clinic, Department of Molecular Medicine, Scripps Research, La Jolla, CA 92037, USA

**Keywords:** discoid lateral meniscus, osteochondral lesion, genu valgum, guided growth, hemiepiphysiodesis, meniscal repair, saucerization, pediatric knee

## Abstract

**Highlights:**

**What are the main findings?**

- A torn discoid lateral meniscus, an ICRS grade 4 osteochondral lesion of the lateral femoral condyle measuring 1.4 × 1.6 cm, and mild valgus alignment (4° on the hip–knee–ankle axis, mechanical axis deviation 10.78 mm lateral) were managed in a single operative episode by saucerization with repair, microfracture, and temporary hemiepiphysiodesis.
- Alignment was corrected to neutral by five months, when the implants were removed, and remained neutral at two years without rebound deformity; the repaired meniscus adopted a more triangular profile with a mid-body width of 9 mm and no extrusion.

**What are the implications of the main findings?**

- Coronal alignment is worth assessing in the growing knee that presents with intra-articular lateral compartment pathology, but the decision to correct it should be individualized rather than driven by the magnitude of the deformity alone.
- Two years is too short to judge joint preservation; the physes were almost closed by then, which makes late rebound unlikely, but the degenerative endpoint depends on loading over decades.

**Abstract:**

**Background/Objectives:** Valgus alignment shifts load toward the lateral compartment, and even a mild shift may matter when that compartment already contains a torn meniscus and an osteochondral lesion. We describe a skeletally immature knee in which a torn discoid lateral meniscus (DLM), an osteochondral lesion of the lateral femoral condyle and mild valgus coexisted. **Case Presentation:** A 14-year-old boy presented with longstanding right knee pain, lateral joint line tenderness and a positive McMurray test. The hip–knee–ankle (HKA) axis showed 4° of valgus (contralateral 0.5°), with a mechanical lateral distal femoral angle (mLDFA) of 84.6°, a medial proximal tibial angle (mMPTA) of 90.0° and mechanical axis deviation (MAD) 10.78 mm lateral. Imaging and arthroscopy showed a torn, centrally shifted DLM and a 1.4 × 1.6 cm ICRS grade 4 lesion of the lateral femoral condyle. Saucerization with repair, microfracture, and temporary hemiepiphysiodesis of the medial distal femoral and proximal tibial physes were performed in one stage. **Results:** At five months, the HKA was 0.7° varus (mLDFA, 87.2°; mMPTA, 88.3°) and the screws were removed; the repaired meniscus measured 9 mm in width without extrusion. At two years, the HKA was neutral (mLDFA, 87.0°; mMPTA, 87.7°; MAD, 0 mm) without rebound, and the patient was asymptomatic; no validated patient-reported outcome measure was collected. **Conclusions:** As a short-term result in a single patient, this case illustrates the feasibility of incorporating temporary hemiepiphysiodesis into a joint-preserving strategy in a selected skeletally immature knee. It is not evidence of efficacy; longer-term follow-up is needed to judge joint preservation.

## 1. Introduction

In the skeletally immature knee, coronal alignment determines how load is shared between the medial and lateral compartments at a stage when articular cartilage, subchondral bone and meniscal tissue are still maturing. Alignment is therefore worth characterizing whenever intra-articular pathology confined to one compartment is being treated.

Discoid lateral meniscus (DLM) is a congenital morphologic variant in the knee that is more prevalent in Asian populations and is vulnerable to tearing and mechanical symptoms because of its abnormal shape, altered ultrastructure, and instability [1,2,3,4]. Current treatment of symptomatic DLM emphasizes meniscal preservation through arthroscopic reshaping with or without repair rather than subtotal or total meniscectomy, because excessive meniscal resection may result in later degenerative change [5,6,7,8]. Magnetic resonance imaging (MRI)-based studies have further shown that the residual meniscus continues to undergo morphological change after surgery, underscoring the importance of preserving adequate meniscal tissue [9,10,11,12].

The lateral compartment is particularly exposed to this effect. Displacement of the mechanical axis alters the share of load carried by each compartment [13], and a valgus shift, even a mild one, directs a greater share laterally. In a knee with an intact lateral compartment, such a shift may be well tolerated. Where the lateral meniscus is discoid, torn and unstable, however, normal load distribution may already be compromised, and where the articular surface carries a full-thickness osteochondral lesion, the additional load falls on a surface that is already damaged. The relevance of mild valgus may therefore depend as much on the state of the compartment toward which it directs load as on its magnitude.

Symptomatic DLM is a familiar entity, and its coexistence with an osteochondral lesion of the lateral femoral condyle and valgus alignment has rarely been described [14,15]. The clinical challenge such a knee presents, however, lies less in its rarity than in the need to address the intra-articular lesions and the coronal alignment together while preserving the growth that remains. Addressing both within the same treatment strategy allows the alignment to be corrected during the period in which the repaired meniscus and the treated osteochondral lesion are healing; whether the procedures are better performed simultaneously or in stages remains uncertain. We report a skeletally immature patient in whom the three findings were addressed in a single operative episode combining arthroscopic saucerization with meniscal repair, microfracture and temporary hemiepiphysiodesis, and we set out the individualized reasoning behind adding guided growth. We do not claim that the three findings are causally linked; a single case cannot establish such a relationship. This report follows the CARE reporting guideline [16].

## 2. Case Presentation

### 2.1. Patient Information

A 14-year-old boy presented with right knee discomfort that had persisted for several years. Two to three weeks before presentation, he developed severe aggravation of pain without any definite history of trauma. He had no relevant past medical history, no prior knee surgery, and no family history of skeletal dysplasia or angular deformity.

### 2.2. Clinical Findings and Diagnostic Assessment

On physical examination, the patient had lateral joint line tenderness, clicking and pain during range of motion, a positive McMurray test, and pain on full extension. Neurovascular examination was normal and there was no effusion or ligamentous instability.

Full-length standing anteroposterior radiographs of both lower extremities demonstrated 4° of valgus of the right limb on the hip–knee–ankle (HKA) axis, against 0.5° of valgus on the unaffected left limb. Deformity analysis localized the valgus principally to the distal femur: the mechanical lateral distal femoral angle (mLDFA) was 84.6°, below the reference range of 85–90°, while the medial proximal tibial angle (mMPTA) was 90.0°, at the upper limit of that range [17]. The mechanical axis deviation (MAD) was 10.78 mm lateral to the knee centre, so the weight-bearing line passed through the lateral compartment. The joint line convergence angle (JLCA) was 0.1°, indicating that soft-tissue laxity made no meaningful contribution and that the deformity was osseous. Alignment parameters at each time point are given in Table 1. Knee radiographs showed an osteochondral lesion of the lateral femoral condyle (Figure 1a,b).

MRI showed a torn DLM with central shifting of the meniscal body and an osteochondral lesion of the lateral femoral condyle measuring approximately 1.4 × 1.6 cm (Figure 2a,b). The physes about the knee were open on radiographs, indicating that growth remained; skeletal age was not formally assessed. Arthroscopy subsequently confirmed a complete discoid lateral meniscus (Watanabe classification [1,3]) that was unstable, corresponding to the central shift type of the magnetic resonance classification based on peripheral attachment [4], together with a full-thickness osteochondral lesion of the lateral femoral condyle graded ICRS 4 [18].

Three findings of the lateral compartment therefore coexisted: a congenital meniscal variant that had torn, a full-thickness osteochondral lesion of the weight-bearing surface, and a mechanical axis displaced toward that surface. They were considered together as a single problem of lateral compartment loading. This framing guided treatment; it is not a demonstrated causal relationship, and the present case cannot establish one.

### 2.3. Therapeutic Intervention

The intra-articular lesions were the primary indication for surgery. The addition of guided growth was an individualized decision rather than treatment of the valgus in its own right. A 4° HKA valgus is within the range usually regarded as physiological in adolescence, and hemiepiphysiodesis is not proposed here as a standard response to mild valgus accompanying DLM. In this patient three considerations were weighed together: the mechanical axis lay 10.78 mm lateral to the knee centre and therefore passed through the compartment containing the lesion; the affected limb differed from the contralateral limb by 3.5°, and the mLDFA of 84.6° was slightly below the reference range, indicating a predominantly distal femoral contribution to the valgus alignment; and the lesion on the loaded surface was full-thickness. The intent was to reduce load across the affected compartment while the intra-articular lesions healed, rather than to correct a deformity of clinical significance in itself. All three components were addressed in a single stage.

Arthroscopic saucerization was combined with meniscal repair to preserve as much functional meniscal tissue as possible while restoring a more normal meniscal contour. The peripheral tear was repaired with four all-inside and two inside-out sutures (Figure 3a,b).

The osteochondral lesion of the lateral femoral condyle was treated with microfracture. A standard arthroscopic awl was used to create perforations approximately 3–4 mm deep and 3–4 mm apart across the exposed subchondral bone (Figure 3c,d).

Temporary hemiepiphysiodesis was then performed with 7.0 mm cannulated screws 105 mm in length (BK Meditech, Seoul, Republic of Korea), inserted percutaneously across the medial distal femoral and the medial proximal tibial physis (Figure 4). Although the deformity was predominantly femoral, the proximal tibial physis was instrumented as well. At 14 years, the remaining growth was limited, and tethering both physes was intended to maximize the correction achievable within that growth rather than to treat a discrete proximal tibial deformity; the mMPTA lay at the upper limit of the reference range rather than outside it.

### 2.4. Postoperative Rehabilitation

Rehabilitation followed the constraints imposed by the meniscal repair and the microfracture, since the hemiepiphysiodesis itself required no protection. The knee was immobilized in a splint for the first two weeks. A brace was applied from the third week, and the range of motion was advanced from an initial arc of 0–60° by approximately 15° each week. Weight-bearing was advanced progressively, and full weight-bearing was reached at eight weeks. Return to sport was allowed between five and six months, once strength and neuromuscular control had been regained.

### 2.5. Outcome and Follow-Up

Follow-up comprised serial radiographic and MRI evaluation. At five months, the HKA measured 0.7° of varus, with an mLDFA of 87.2°, an mMPTA of 88.3° and the mechanical axis 1.2 mm lateral to the knee centre (Table 1). The correction obtained was 2.6° at the distal femur and 1.7° at the proximal tibia. The screws were removed at that visit because neutral alignment had been reached while the physes were still open; removal before physeal closure was intended to avoid overcorrection. No tethering, asymmetric physeal narrowing or premature closure was seen on radiographs obtained before or after removal, and radiographs showed reduced radiolucency at the site of the osteochondral lesion (Figure 5a,b).

MRI at five months showed the repaired meniscus to have adopted a more triangular profile on coronal images, with a mid-body width of 9 mm and no extrusion (Figure 5c). Signal within the repaired segment had decreased relative to the preoperative study. These are descriptions of the imaging appearance. The contralateral meniscus was not imaged, no second-look arthroscopy was performed, and quantitative cartilage assessment was not undertaken, so neither meniscal healing nor cartilage repair can be confirmed from these findings.

At the latest follow-up, two years after surgery, the patient reported no pain or mechanical symptoms and had returned to unrestricted activity. The HKA was neutral, with an mLDFA of 87.0°, an mMPTA of 87.7° and the mechanical axis passing through the knee centre; no rebound valgus had developed in the nineteen months after screw removal (Figure 6a,b). Radiographs showed further reduction in the radiolucency at the lateral femoral condyle, and the physes about the knee were by then almost completely closed, indicating that little further growth remained. No validated patient-reported outcome measure was collected at any point, so the clinical result is reported qualitatively.

## 3. Discussion

The feature of interest in this case is the coexistence of a torn DLM, a full-thickness osteochondral lesion of the lateral femoral condyle and lateral deviation of the mechanical axis in a skeletally immature knee. Treatment confined to the meniscus would have left the other two findings unaddressed, and the approach taken was therefore directed at the lateral compartment as a whole. Whether the three findings are causally related cannot be determined from a single patient. They may share a mechanical origin, or the alignment may simply have coexisted. What the case shows is that all three could be addressed in one operative episode and that the alignment correction was achieved and maintained over two years.

Even in the presence of an extensive tear, meniscal preservation should be attempted whenever feasible [19]. Contemporary treatment of symptomatic DLM favours saucerization with repair over extensive meniscectomy, because functional meniscal tissue is considered important for long-term joint protection [20,21], and MRI-based studies have shown that the residual meniscus may narrow and continue to remodel after reshaping surgery [9,10,11,14]. In the present case, the repaired meniscus adopted a more triangular profile, with a mid-body width of 9 mm and no extrusion at five months. This is a morphological observation. It does not establish that meniscal load transmission returned to normal, and without contralateral imaging or second-look arthroscopy the integrity of the repair can only be inferred from the reduction in signal within the repaired segment.

The rationale for correcting alignment rests on the relationship between coronal deformity and lateral compartment degeneration. Progression to valgus alignment and lateral compartment degeneration has been reported after surgery for adult DLM [22], which suggests that alignment is not simply an incidental finding in these knees. The valgus here was nonetheless mild by the usual thresholds, and we would not propose hemiepiphysiodesis for every adolescent with mild valgus and a DLM. The decision was individualized and compartment-protective: the mechanical axis passed lateral to the knee centre, the mLDFA lay slightly below the reference range, the affected limb differed from the contralateral limb by 3.5°, and the lesion on the loaded surface was full-thickness.

In a skeletally immature patient, remaining growth offers a means of correcting deformity without osteotomy, and temporary hemiepiphysiodesis was selected on that basis [23]. At 14 years, however, remaining growth is limited, which constrains both the magnitude and the predictability of the correction achievable by guided growth; both physes were tethered in order to maximize the correction available within that growth, rather than because the proximal tibia harboured a discrete deformity. Correction to neutral was reached within five months, and the implants were removed as soon as it was documented, before physeal closure, in order to avoid overcorrection. No rebound occurred over the following nineteen months, and by the two-year review, the physes were almost completely closed, leaving little growth through which a recurrent deformity could develop. Rebound remains a recognized concern after guided growth, particularly in younger patients with more growth remaining, but in this patient the window in which it could have arisen has largely passed.

Correcting the mechanical axis may also bear on the environment in which the intra-articular lesions heal. Moving the load-bearing line from 10.78 mm lateral to the knee centre reduces the share of load carried by the lateral compartment [13], and it does so during the months in which the repaired meniscus and the repair tissue expected after microfracture are maturing. Whether this unloading improves the quality or durability of repair cannot be determined from the present case. It is offered as a theoretical rationale for combining alignment correction with joint-preserving procedures, not as a demonstrated effect.

For a clinician encountering a similar knee, the factors weighed in this case may serve as considerations rather than criteria: the position of the mechanical axis relative to the compartment containing the lesion; whether the deformity lies outside reference ranges or differs from the contralateral limb; the severity of the intra-articular pathology; and the growth that remains. No threshold for any of these can be derived from a single case, and none is proposed.

The lesion in the present case was characterized descriptively, by its dimensions and its arthroscopic ICRS grade, without attempting to assign a specific etiology. What distinguishes the present case is that all three findings were present at first presentation and were managed within a single operative episode.

This report has several limitations. It describes a single patient, and the relative contributions of saucerization, meniscal repair, microfracture and hemiepiphysiodesis cannot be separated, nor can the case establish that the three findings were causally related. Skeletal age was not formally assessed, so remaining growth was judged from the radiographic appearance of the physes rather than estimated quantitatively. No validated patient-reported outcome measure was collected, and no dynamic gait assessment was performed, so the clinical and functional result is described qualitatively. The imaging interpretation is limited in the same way: quantitative cartilage assessment was not performed, the contralateral meniscus was not imaged for comparison, and no second-look arthroscopy was undertaken, so the radiographic and MRI changes describe appearance rather than confirmed tissue repair.

Most importantly, two years is too short to address the endpoint that matters. Joint preservation is judged by the absence of lateral compartment degeneration over a much longer horizon, and in a patient operated at 14 years, this cannot be assessed before skeletal maturity, particularly since degeneration may progress over time even after meniscus-preserving surgery for DLM [19,20]. The near-complete physeal closure seen at two years makes late recurrence of the deformity unlikely, but skeletal maturity and the degenerative endpoint are separate questions: the latter depends on loading over decades rather than on remaining growth. The findings reported here should therefore be read as short-term feasibility rather than as evidence of joint preservation, and longer-term surveillance of the lateral compartment remains necessary. Future work in larger series could incorporate three-dimensional motion analysis and dynamic assessment of joint loading to characterize how knee mechanics change after alignment correction.

## 4. Conclusions

As a short-term result in a single patient, this case illustrates the feasibility of incorporating temporary hemiepiphysiodesis as an adjunct into a joint-preserving strategy in a selected skeletally immature patient with concomitant discoid lateral meniscus, osteochondral pathology and mild valgus alignment. It should be regarded as an illustrative proof-of-concept rather than a generally recommended treatment approach; larger case series or comparative studies would be needed to establish efficacy and define appropriate indications. Hemiepiphysiodesis is not proposed as a standard response to mild valgus accompanying DLM: the indication here was individualized and driven by the combination of a laterally deviated mechanical axis and a full-thickness osteochondral lesion on the loaded surface, rather than by the magnitude of the valgus. Guided growth may be considered in selected skeletally immature patients with coronal malalignment and sufficient remaining growth, when alignment is judged relevant to the mechanical environment of the affected compartment; this case does not establish an indication for it. The correction was maintained through the two-year follow-up, and with the physes almost closed at that time, the risk of substantial late rebound appears limited. What remains unknown is whether this strategy protects the lateral compartment over the long term, and that question cannot be answered at two years. What the case does suggest more generally is that coronal alignment deserves assessment in the growing knee presenting with intra-articular pathology of the lateral compartment.

## Figures and Tables

**Figure 1:**
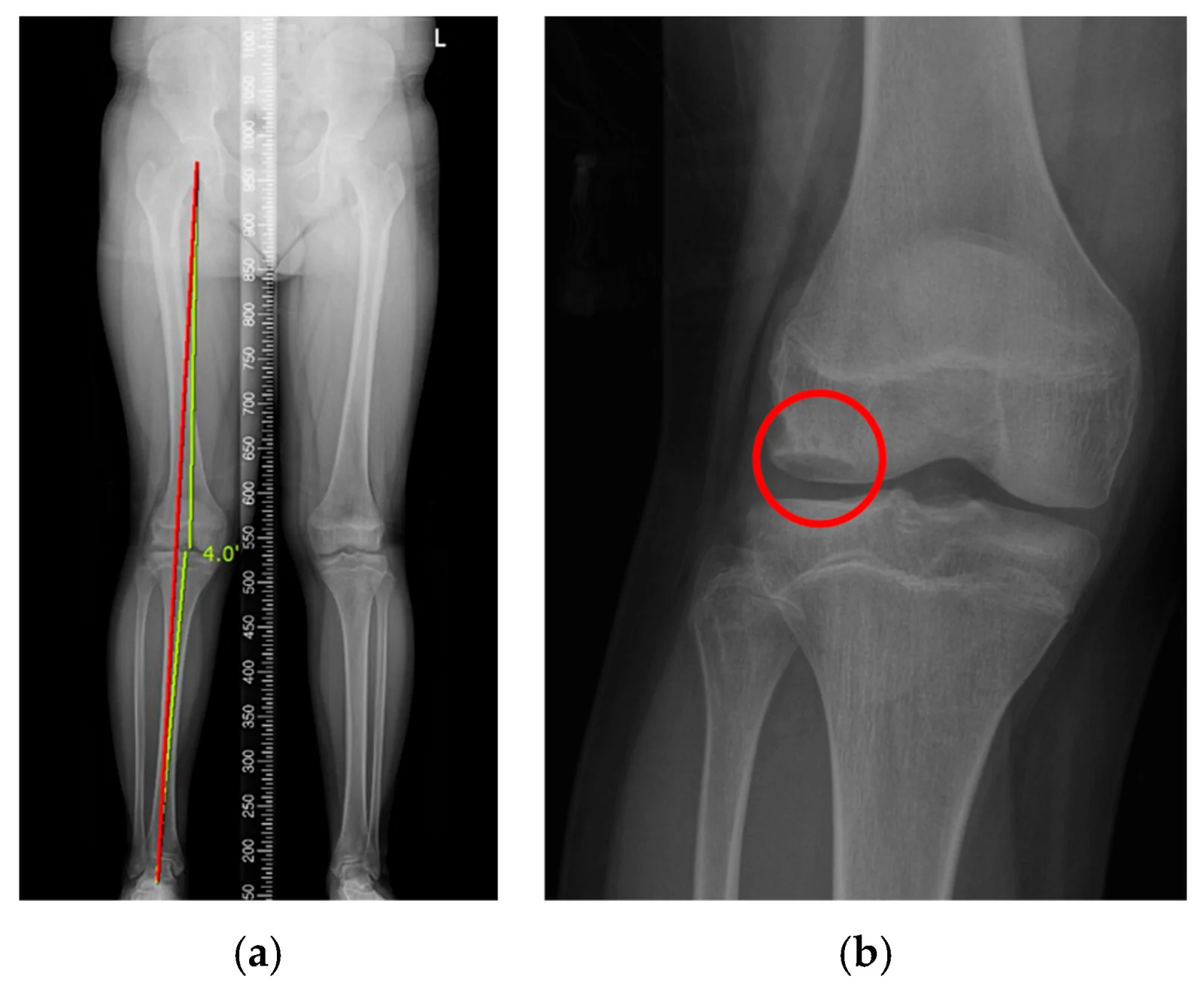
Preoperative radiographs of the right lower extremity. (**a**) Whole-leg full-length standing anteroposterior radiograph demonstrating 4° valgus alignment on the hip–knee–ankle axis (green lines); the red line is the mechanical weight-bearing axis. (**b**) Anteroposterior radiograph of the right knee demonstrating an osteochondral lesion of the lateral femoral condyle (red circle).

**Figure 2:**
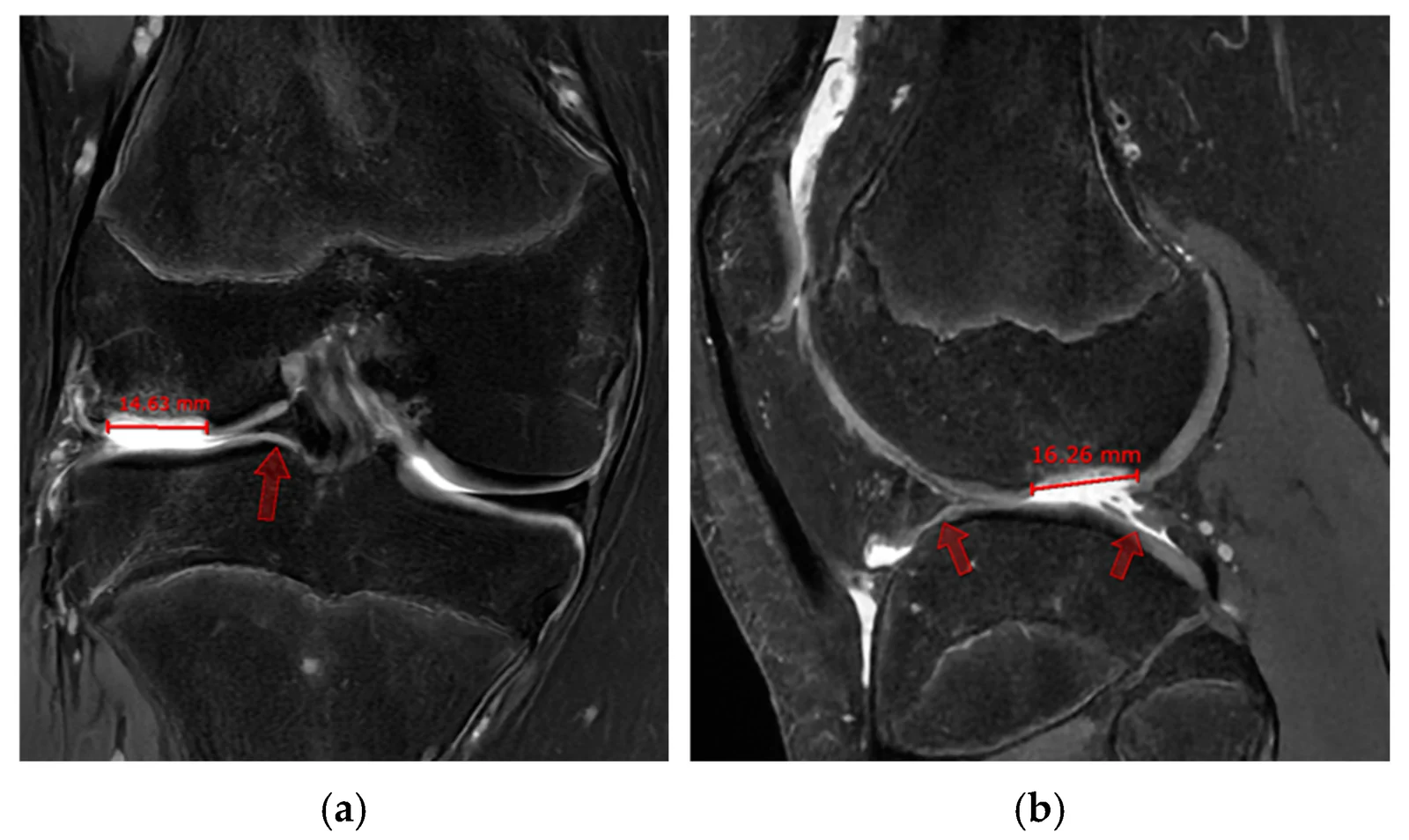
Preoperative magnetic resonance images of the right knee. (**a**) Coronal and (**b**) sagittal images demonstrating a torn discoid lateral meniscus with meniscal shifting and an osteochondral lesion of the lateral femoral condyle measuring approximately 1.4 × 1.6 cm. Red arrows indicate the torn discoid lateral meniscus with meniscal shifting.

**Figure 3:**
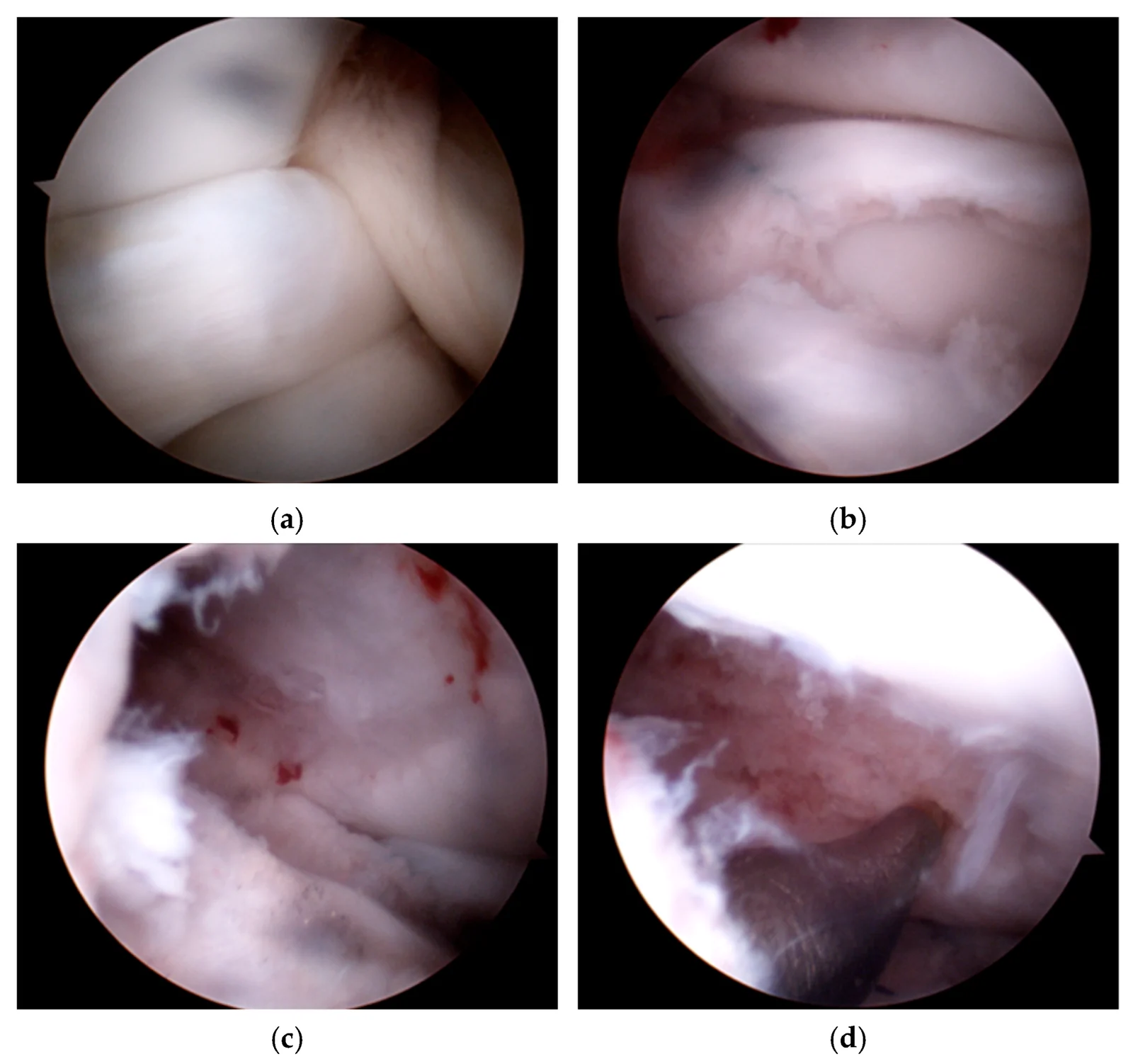
Arthroscopic findings and treatment of the right knee. (**a**) Arthroscopic view confirming a complete and unstable discoid lateral meniscus. (**b**) Overall arthroscopic appearance after reshaping surgery. (**c**,**d**) Arthroscopic microfracture performed for the osteochondral lesion of the lateral femoral condyle.

**Figure 4:**
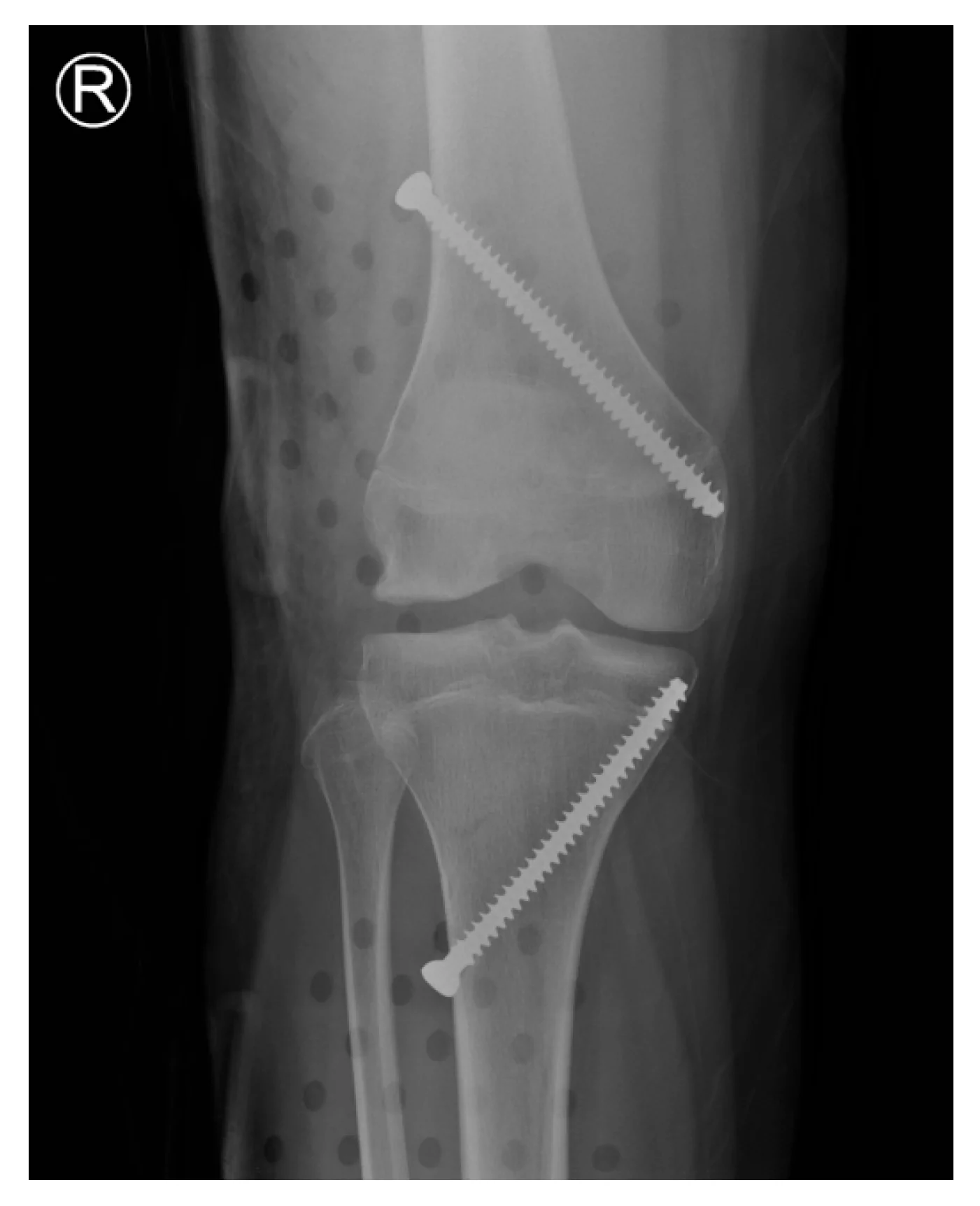
Postoperative radiograph showing temporary hemiepiphysiodesis using percutaneous epiphysiodesis transphyseal screws across the medial distal femoral and medial proximal tibial physes for correction of valgus malalignment.

**Figure 5:**
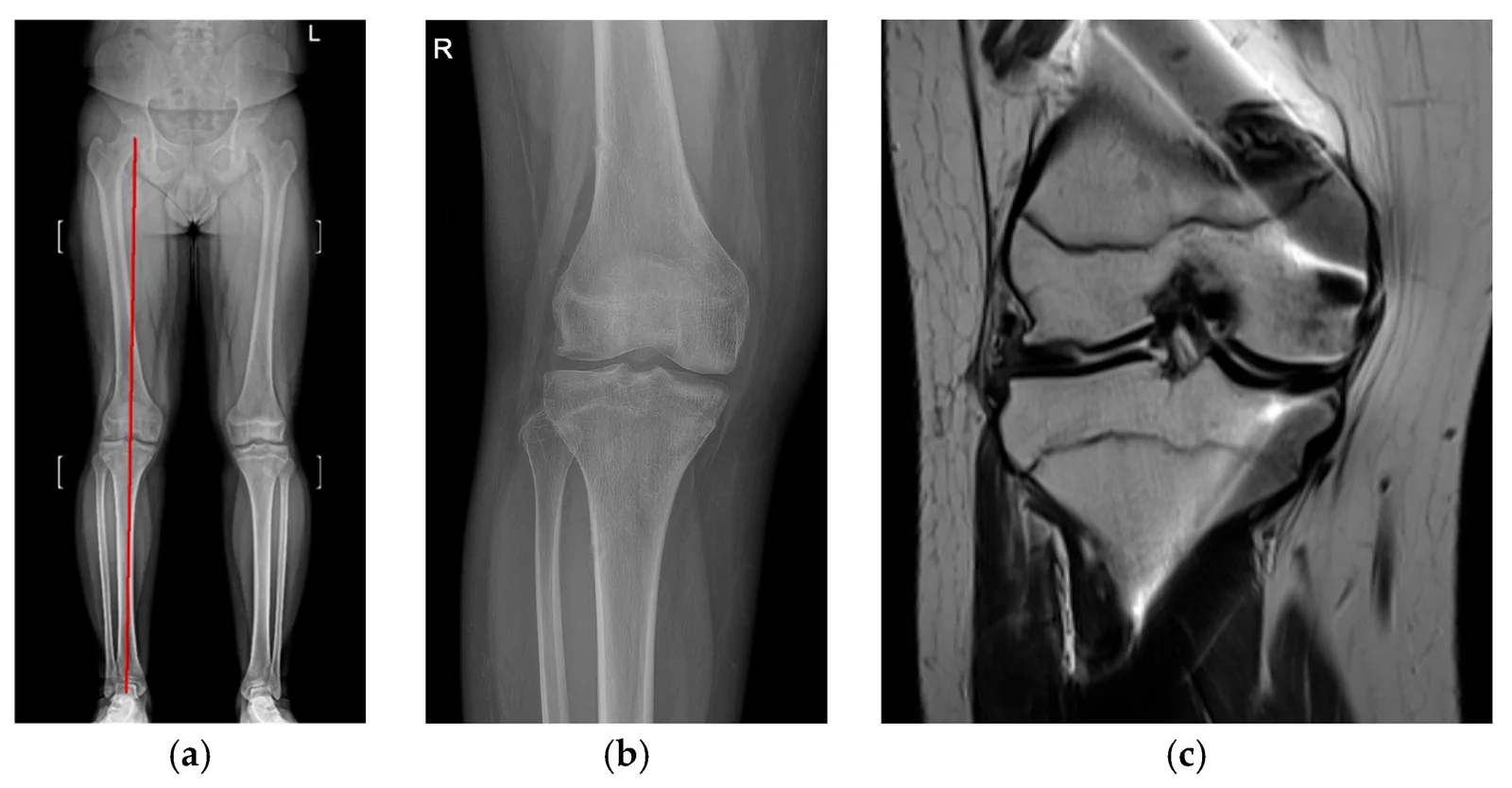
Follow-up imaging at five months after surgery. (**a**) Whole-leg full-length standing anteroposterior radiograph demonstrating correction of lower-extremity alignment to neutral (red line). (**b**) Anteroposterior radiograph of the right knee demonstrating decreased radiolucency of the previous osteochondral lesion of the lateral femoral condyle. (**c**) Follow-up magnetic resonance image showing a more triangular configuration of the repaired discoid lateral meniscus and postoperative changes at the site of the osteochondral lesion.

**Figure 6:**
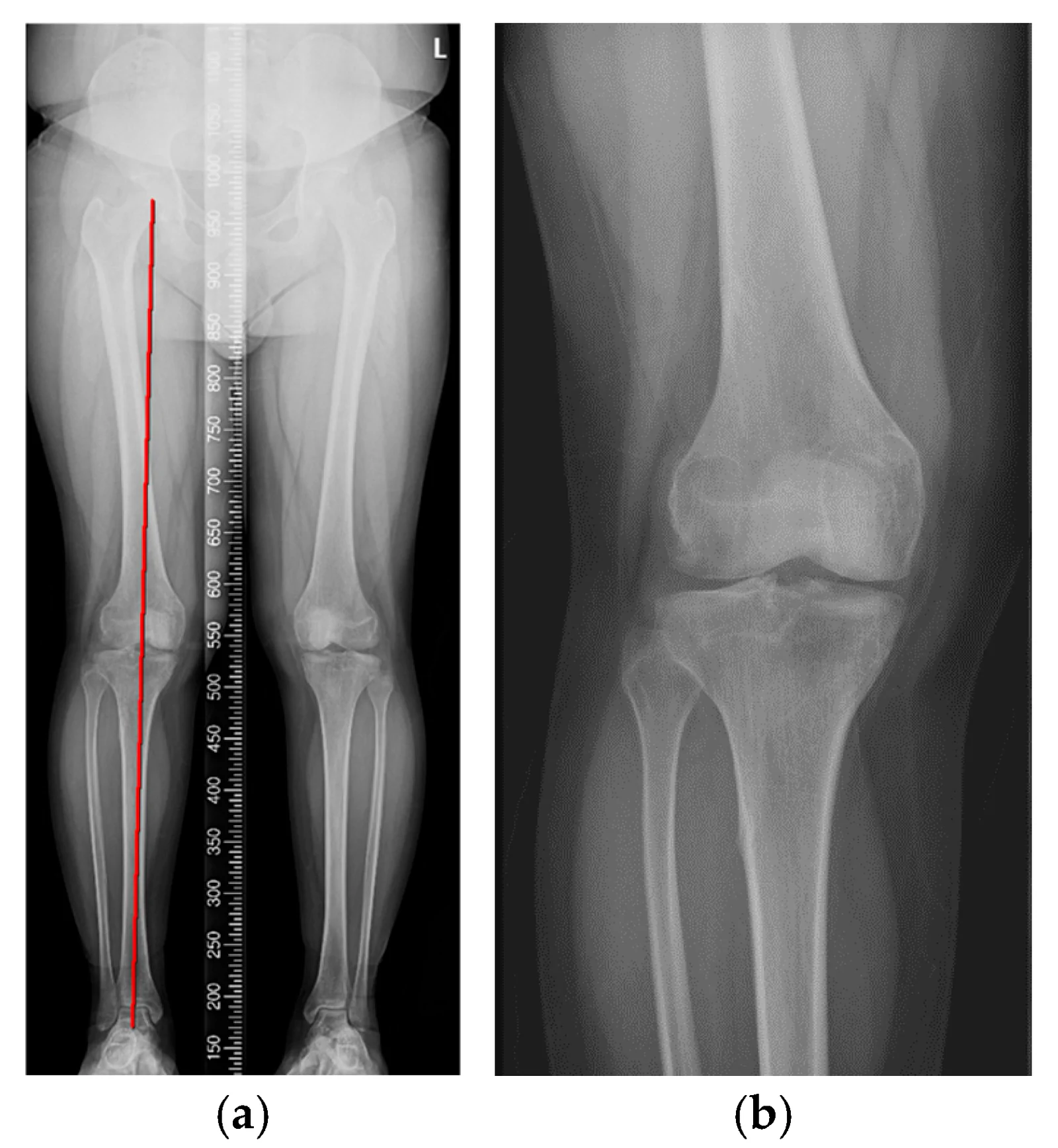
Radiographs at two-year follow-up. (**a**) Whole-leg full-length standing anteroposterior radiograph demonstrating maintained neutral alignment without rebound deformity after screw removal (red line). (**b**) Anteroposterior radiograph of the right knee showing further reduction in the radiolucency at the site of the osteochondral lesion of the lateral femoral condyle.

**Table 1:** Coronal plane alignment parameters of the affected right limb before surgery, at five months and at two years, with the contralateral limb for reference.

Parameter	Preoperative	5 Months	2 Years	Contralateral
HKA (°, valgus positive)	4.0	−0.7	0.0	0.5
mLDFA (°)	84.6	87.2	87.0	n.m.
mMPTA (°)	90.0	88.3	87.7	n.m.
JLCA (°)	0.1	0.1	0.2	n.m.
MAD (mm, lateral positive)	10.78	1.2	0.0	n.m.

HKA, hip–knee–ankle angle; mLDFA, mechanical lateral distal femoral angle; mMPTA, medial proximal tibial angle; JLCA, joint line convergence angle; MAD, mechanical axis deviation; n.m., not measured. Negative HKA values denote varus. Reference ranges: mLDFA, 85–90°; mMPTA, 85–90°; JLCA, 0–2° [17].

## Data Availability

The data presented in this study are available in the article.

## Abbreviations

The following abbreviations are used in this manuscript:

DLM	Discoid lateral meniscus
HKA	Hip–knee–ankle
MRI	Magnetic resonance imaging
ICRS	International Cartilage Repair Society
JLCA	Joint line convergence angle
MAD	Mechanical axis deviation
mLDFA	Mechanical lateral distal femoral angle
mMPTA	Medial proximal tibial angle
